# The costs of headache disorders

**DOI:** 10.1186/1129-2377-16-S1-A3

**Published:** 2015-09-28

**Authors:** Francesco Saverio Mennini, Lara Gitto

**Affiliations:** CEIS, EEHTA, University Tor Vergata, Rome, Italy; Institute for Leadership and Management, Kingston University, London, UK

The impact of headache disorders is a problem of enormous proportions, both for the individual and the society. Medical literature has tried to assess its effects on individuals, by examining prevalence, distribution, attack frequency and duration, and headache-related disability, as well as effects on society, looking at the socio-economic burden of headache disorders.

The issue of costs represents an important problem too, concerning both direct and indirect costs. Direct costs concern mainly expenses for drugs.

Migraine has a considerable impact on functional capacity, resulting in disrupted work and social activities: many migraineurs do not seek medical attention because they have not been accurately diagnosed by a physician or do not use prescribed medication[1].

Indirect costs associated with reduced productivity represent a substantial proportion of the total cost of migraine as well. Migraine has a major impact on the working sector of the population, and therefore, determining the indirect costs outweighs the direct costs. This study will explain the notion of cost of illness, examining how it could be applied in such a framework. Then, an overview of the studies aimed at measuring direct and indirect costs of migraine and headache disorders will be carried out, later shifting on to the relationship between costs and quality of life for people affected by headache disorders.

As it has been seen, there are still many unsolved problems in disease costing, to the point that it still appears as a set of method that may lead to extremely different outcomes depending on the evaluation approach being used. Moreover, given the social relevance of migraine, together with the assessment of therapeutic options, it is important to increase the knowledge related to the economic consequences of prevention. From the analysis of prevalence, incidence, morbidity and the state of health caused by headache, it is important to stimulate the scientific community and policy makers to analyze the problems connected to the economic costs of headache. Costs of headache could be contained by observing their trends implementing specific “observatories”.

Overall, the bottom-up approach, applied in the Eurolight study, would seem the preferable and most comprehensive method to assess the societal burden of headache. However, a crucial factor is the attainment of a higher participation to the survey.
